# Acute appendicitis caused by acute myeloid leukemia

**DOI:** 10.1002/ccr3.89

**Published:** 2014-09-15

**Authors:** Shanxiang Zhang, Shaoxiong Chen

**Affiliations:** Department of Pathology and Laboratory Medicine, Indiana University350 West 11th Street, Indianapolis, Indiana, 46202

**Keywords:** Abdominal pain, acute appendicitis, acute myeloid leukemia, myelodysplastic syndrome

## Abstract

**Key Clinical Message:**

A case of appendiceal involvement by acute myeloid leukemia (AML) in an adult with recent history of AML transformed from myelodysplastic syndrome (MDS) was presented. Being aware of this rare presentation in particular in a patient with history of MDS and/or AML is important for prompt clinical diagnosis and management.

A 57-year-old man with a seven-month history of refractory anemia with excess blasts (RAEB-2), recently transformed to acute myeloid leukemia (AML) presented with a 3-day history of lower abdominal pain, nausea, and constipation. There was no fever or vomiting. Physical examination revealed tenderness in his abdominal bilateral lower quadrants. CAT scan revealed a thickened appendix and periappendiceal stranding. The liver, spleen, pancreas, adrenal glands, and kidneys were normal. The gall bladder was previously removed and was not visible. His peripheral blood count showed white blood cell count of 2.3 × 10^9^/L with granulocytic left shift and few blasts (4%), hemoglobin of 8.3 g/dL, and platelet count of 34 × 10^9^/L. An acute appendicitis was suspected and a laparoscopic appendectomy was performed. Visual inspection of the peritoneal cavity during the procedure was essentially unremarkable. The appendix appeared to be inflamed and grossly showed no adhesions. Histologic sections (Figure 1) revealed extensive proliferation of hematopoietic cells involving predominantly serosa and periappendiceal tissue. There were clusters and aggregates of large immature precursor cells with irregular nuclear contours, delicate chromatin, distinct nucleoli, and scant to moderately abundant cytoplasm. Rare islands of erythroid precursor cells were present. The mucosa, submucosa, and muscularis propria of the appendix were histologically unremarkable. Immunohistochemical stains showed the large precursor cells were weakly positive for myeloperoxidase antigen, and partially positive for CD68 (PGM-1) and CD163. Scattered CD34- and/or CD117-positive blasts were present, though majority of the large precursor cells were negative. Few lymphoid aggregates containing mixed CD20-positive B cells and CD3-positive T cells were noted. A diagnosis of appendix involved by AML was rendered.

Acute appendicitis due to involvement by acute leukemia is exceedingly rare 1–3. Being aware of this possibility is very important in the prompt diagnosis and management of these patients who may present symptoms mimicking acute appendicitis.

**Figure 1:**
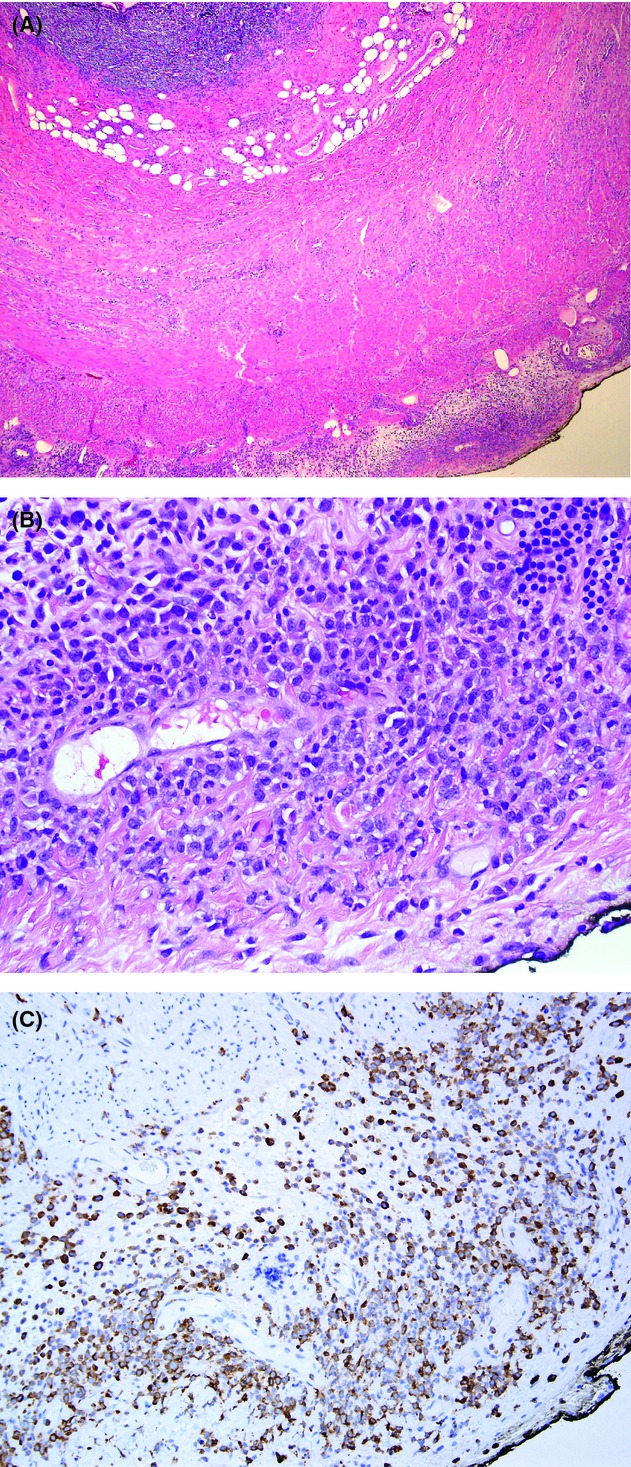
(A) Cellular infiltration involving serosa and periappendiceal tissue. Hematoxylin and eosin, 4×. (B) Hematopoietic cells with sheets of large immature precursor cells. Hematoxylin and eosin, 40×. (C) Immunohistochemical stain for myeloperoxidase.
